# Cyclooxygenase-2 (COX-2) Expression in Equine Melanocytic Tumors

**DOI:** 10.3390/vetsci11020077

**Published:** 2024-02-07

**Authors:** José Pimenta, Justina Prada, Isabel Pires, Mário Cotovio

**Affiliations:** 1 CECAV—Veterinary and Animal Research Center, University of Trás-os-Montes e Alto Douro, 5000-801 Vila Real, Portugal; jprada@utad.pt (J.P.); ipires@utad.pt (I.P.); mcotovio@utad.pt (M.C.); 2 Associate Laboratory for Animal and Veterinary Sciences (AL4AnimalS), 5000-801 Vila Real, Portugal; 3 CIVG—Vasco da Gama Research Center, EUVG—Vasco da Gama University School, 3020-210 Coimbra, Portugal; 4 Veterinary Sciences Department, University of Trás-os-Montes e Alto Douro, 5000-801 Vila Real, Portugal; 5 Faculty of Veterinary Medicine, Lusófona University, Campo Grande 376, 1749-024 Lisbon, Portugal

**Keywords:** equine, melanocytic tumors, COX-2

## Abstract

**Simple Summary:**

Cyclooxygenase-2 (COX-2) has been associated with melanoma progression in humans and dogs. Its overexpression is related to tumor aggressiveness. Equine melanoma has a different pattern of evolution compared to that in dogs and humans since it is characterized by a slow, expansive growth rather than a high degree of invasiveness and frequent metastasis. The aim of this study was to evaluate the immunohistochemical expression of COX-2 in equine melanocytic tumors. Immunohistochemistry was used to evaluate 39 melanocytomas and 38 melanomas. The findings indicated that 42.9% of melanocytic tumors were negative, 41.6% had low COX-2 expression, and 15.5% had high COX-2 expression. Meanwhile, 13.2% of malignant tumors were negative and 63.2% presented low COX-2 expression. COX-2 was significantly higher in melanomas than in melanocytomas. Overall, low COX-2 levels may be one of the molecular differences that may contribute to the different clinical behavior of equine melanocytic tumors compared with those of other species.

**Abstract:**

Equine melanocytic tumors are common and have an unusual benign behavior with low invasiveness and metastatic rates. However, tumoral mass growth is usually a concern that can have life-threatening consequences. COX-2 is related to oncogenesis, promoting neoplastic cell proliferation, invasion, and metastasis. The aim of this study was to evaluate the immunohistochemical expression of COX-2 in equine melanocytic tumors. Through extension and intensity of labeling, 39 melanocytomas and 38 melanomas were evaluated. Of the malignant tumors, 13.2% were negative and 63.2% presented a low COX-2 expression. Only 6 malignant tumors presented >50% of labeled cells, 18 malignant and 8 benign had an expression between 21 and 50%, 8 malignant and 3 benign tumors had an expression between 6 and 20%, 1 malignant tumor had an expression between 1 and 5%, and 5 malignant and 28 benign tumors had no expression. Malignant tumors showed higher COX-2 expression than did benign tumors, with statistically significant differences. The low levels of COX-2 may be one of the molecular reasons for the presence of expansive mass growth instead of the invasive pattern of other species, which is related to high COX-2 levels.

## 1. Introduction

Melanocytic tumors are one of the most concerning oncological diseases in both human and veterinary medicine, being the third most common tumor type in horses [1,2,3,4]. Although this tumor type is transversal to both areas, some clinical differences exist between humans and the commonly affected domestic species, dogs and horses [2,3,4]. In humans, melanoma is an aggressive cancer that is able to kill thousands of people worldwide [1,4,5,6]. The cutaneous form is the most common, being responsible for approximately 65% of all skin-malignancy-related deaths. Clinically, it is characterized as being highly aggressive, showing marked vertical invasion and frequent metastasis formation [1,4,5,6]. Canine melanocytic tumors are also common, with the clinical course being related to tumor localization. Oral and mucocutaneous forms are usually highly malignant and invasive and often metastasize early in the disease course, being similar to human melanoma [2,7,8]. On the other hand, the cutaneous form more often presents a benign behavior.

In contrast, equine melanocytic tumors have a very distinct clinical course. They usually emerge on cutaneous and mucocutaneous localizations such as the perianal region, tail, lips, eyelids, genital area, and parotid region, with a higher prevalence in gray horses due to genetic predisposition [9,10,11,12,13]. Even when possessing histological features of malignancy, these tumors tend to present a prolonged benign behavior, distinguished by slow mass expansion, with invasion and metastasis being rarely reported [3,13,14,15,16]. The major clinical problem that clinicians face with equine melanocytic tumors is the massive dimensions that these tumors can acquire, which compromises a few physiological functions and the integrity of some structures due to their being typically located in sensitive areas [15,17]. The size and the location also impair the success of surgical excision, which is currently the most common therapy for equine melanocytic tumors [15,17]. Despite their mostly benign behavior, it has been reported that, in some cases, the clinical behavior of these tumors can change with time, becoming more aggressive. This type of behavior has also been reported to be more common in nongray horses and in amelanotic melanomas [13,16,18,19,20].

Cyclooxygenase (COX) plays an important role in the synthesis of physiologically important prostaglandins. There are three isoforms of COX, namely COX-1, COX-2, and COX-3. COX-1 is constitutively expressed in many tissues under physiological conditions. COX-3 is the less-studied type, being physiologically expressed in the brain and heart. COX-2 is also responsible for the generation of several prostanoids, such as prostaglandin-E2 (PGE2), which is an important mediator of inflammation [21]. The presence of COX-2 is negligible in most normal tissues, with some exceptions, such as stomach, central nervous system, kidney, and female reproductive organs [22]. In contrast to the other isoforms, COX-2 is associated with pathological conditions such as inflammation and carcinogenesis [23,24].

Increased levels of COX-2 expression have already been reported in several human and canine tumors, including melanocytic tumors [21,22,25]. Both COX-2 and PGE2 overexpression account for diverse mechanisms that are linked to tumor initiation and progression in several ways [26]. These include promoting the limitless replication of tumor cells; downregulating adhesion molecules, which leads to tumor cell detachment from the primary tumor mass; promoting tumor invasion through an increase in extracellular matrix degradation; and enhancing neovascularization, a crucial requirement for vascular invasion and tumor spread [21,22,26,27,28,29,30,31,32]. Furthermore, COX-2 also has an immunosuppressive effect, creating a propitious microenvironment for tumor maintenance and progression [21,25,33]. The literature regarding COX-2 expression in equine tumors is scarce, with most studies focusing on equine squamous cell carcinomas [34,35,36,37,38,39,40,41,42]. According to the authors’ knowledge, there is only one report about COX-2 in equine melanoma.

Given all the existent evidence about the role of COX-2 in melanoma biology, the therapeutical inhibition of this biomarker may be an interesting field of research. Both in vitro and in vivo studies have already been performed in human melanoma and indicate that COX-2 inhibitors can be a valuable adjunctive therapy for managing this tumor, contributing to the reduction of the proliferation and invasiveness of tumor cells and the inhibition of metastasis development [23,24,43,44]. According to the author’s knowledge, only in vitro studies exist regarding COX-2 inhibitors’ effect on canine melanoma cancer cells, with most studies mentioning that they might have the potential to be an effective therapeutical option [31,33]. In horses, there is only one case report that mentions the use of a nonsteroidal anti-inflammatory drug (NSAID), piroxicam, for blocking the effect of COX-2 on equine squamous cell carcinoma [40]. Full remission of primary tumors was observed, and remission of the associated metastasis also occurred.

Considering the abundance of knowledge about COX-2, it is important to understand its relevance in equine melanocytic tumors. As such, this work aimed to study the immunohistochemical expression of COX-2 in equine melanocytic tumors, with the aim of correlating it with their atypical behavior and determining whether it can be used as a potential future therapeutical target. We also sought to evaluate the possible associations between COX-2 and the clinicopathological features of these tumors.

## 2. Materials and Methods

### 2.1. Tissue Samples

Formalin-fixed, paraffin-embedded tissue samples of primary cutaneous or mucocutaneous equine melanocytic tumors (collected between 2010 and 2021), with a previous clinical and histological diagnosis, were included in this study. All samples were fixed in 10% formalin for 48 h before processing.

### 2.2. Clinical Information

Information regarding gender, breed, coat color, and mass location was collected although not all clinical records had this information. Age was divided into three categories: young horses were considered those younger than 5 years old, adult horses were those between 6 and 14 years, and geriatric horses were those older than 15 years. To evaluate differences in COX-2 expression, tumors were also divided into cutaneous and mucocutaneous location.

### 2.3. Histopathological Evaluation

For histopathological evaluation, 3 µm sections were stained with hematoxylin and eosin (HE) and re-examined by two independent pathologists (IP, JP), with and without performing a previously described bleaching protocol [45], since melanin pigment impairs the evaluation of some histological features.

For the histological classification (melanocytoma (benign) vs. melanoma (malignant)) the following histological features were used: tumor vascular emboli (present, absent), nuclear grade (I—when nuclei had minimal variations in shape and size compared to normal nuclei; II—moderate alterations on nuclear shape; III—irregular and larger than normal nuclei), and mitotic count (mitosis per ten high power fields (HPF)). Tumors were classified as malignant if presenting the following: (i) tumor vascular emboli, (ii) more than 10 mitoses in 10 HPF, or (iii) fewer than 10 mitoses but a nuclear grade of II or III. For statistical purposes, mitotic count was also divided into absent (0 mitosis) and present (<10 or >10 mitosis).

Tumors were eliminated if they did not resist bleaching or if a reliable histological classification could not be obtained.

The histological evaluation also considered other histological features, including presence of epidermal ulceration (absent, present), circumscription (absent, present), degree of pigmentation (absent, slight, medium, high, very high), and cell shape (epithelioid, spindle, mixed).

### 2.4. Immunohistochemistry

For immunohistochemistry (IHQ), 3 µm sections were mounted on silane-coated slides. Immunolabeling was carried out with a commercial detection system (NovoLink Polymer Detection System; Novocastra, Leica Biosystems, Newcastle, UK) according to the manufacturer’s instructions. Briefly, tissue sections were dewaxed in xylene and hydrated through a decreasing series of alcohol concentrations ending in tap water. Microwave was used for antigen retrieval (3 cycles of 5 min at 750 W) with citrate buffer solution (0.01 M pH 6.0 ± 2). The bleaching protocol was performed after the slides had cooled. Slides were washed in phosphate-buffered saline (PBS) solution; endogenous peroxidase blocking was performed through incubation with 3% hydrogen peroxide for 5 min, and endogenous protein blocking was performed for 5 min. After blocking was completed for nonspecific binding, primary monoclonal antibody anti-COX-2 (clone SP21, Thermo Scientific™ Lab Vision™, Waltham, MA, USA) diluted 1:40 in PBS was incubated in a humidified chamber at 4 °C overnight. Accordingly to the manufacturer, this antibody has already been used in several species including the horse. Slides were washed with PBS for 10 min and incubated with secondary antibody. 3,3′-diaminobenzidine tetrahydrochloride (DAB) chromogen was used for immunolabeling visualization. Slides were counterstained with Gill’s hematoxylin and cover slipped.

### 2.5. Immunohistochemical Evaluation

COX-2 immunolabeling was evaluated blindly and semiquantitatively by two independent pathologists (IP, JP), determining the extent of labeling (percentage of positive cells), the labeling intensity, and labeling localization. Positivity was indicated by a brown membranous and/or cytoplasmatic labeling. Positive control (equine kidney) (Figure S1) and negative control (omission of primary antibody) were included in each staining run.

Labeling extension and intensity were scored according to an adaptation of the methodology followed by [34] as follows: extension—(0) negative, (1) 1–5%, (2) 6–20%, (3) 21–50%, and (4) >50%; intensity of labeling—(0) negative, (1) weak, (2) moderate, and (3) strong. A final immunohistochemical score (IHS) was calculated by multiplying the extension by the intensity of labeling, resulting in a value between 0 and 12.

To categorize tumors with low and high expression, we adapted the methodology used by [46], creating a final score. Low COX-2 expression in tumors was indicated by an immunohistochemical expression score ≤6, while high COX-2 expression in tumors was indicated by an immunohistochemical expression score >6.

Furthermore, we also separated tumors in terms of COX-2 classification into two categories: negative, IHS = 0; and positive, IHS ≥ 1.

### 2.6. Statistical Analysis

To evaluate if COX-2 expression was associated with histological classification or with any histological/clinical features studied, the chi-squared (X^2^) test of independence and Fisher exact test were used. The odds ratio was also determined to evaluate the association between histological classification and COX-2 expression. The Mann-Whitney U test was used to compare medians of labeling extension and intensity between groups (benign and malignant; cutaneous and mucocutaneous). All the results were considered significant when *p* < 0.05. The analyses were performed using Jamovi (version 2.3.2) statistical software.

## 3. Results

### 3.1. Clinical Information

The study sample included 57 horses and a total of 77 melanocytic tumors since two horses presented more than one tumoral mass. Clinical information of some horses was not available. Regarding age, the average age was 14.3 ± 5.44 years, with the youngest being 2 years old and the oldest 26 years old. Regarding age category, 3 horses were young (≤5 years old), 13 were adult (between 6 and 14 years old), and 39 were geriatric (≥15 years old). Regarding gender, there were 28 were males and 27 females. The most common coat color was gray (n = 46) followed by cremello (n = 2), buckskin (n = 1), and brown (n = 1). Affected breeds were purebred Lusitano (n = 27), crossbreed (n = 19), Arabian (n = 3), and warmblood (n = 2). The tumoral masses were distributed along the perianal region (n = 27), tail (n = 32), lips (n = 2), proximal limb (n = 2), trunk (n = 1), vulva (n = 1), neck (n = 1), and parotid gland (n = 1). According to the existing clinical information, 36 tumors were considered cutaneous and 30 mucocutaneous.

### 3.2. Histopathologic Results

According to the histological features mentioned above, the 77 melanocytic tumors were classified as melanocytomas (n = 39) and melanomas (malignant) (n = 38). Regarding the horses with multiples tumoral masses, one horse had 9 melanomas in the perianal region, and the other had 13 melanocytomas on the tail.

Histological classification did not have any significant association with the degree of pigmentation (*p* = 0.26), ulceration (*p* = 0.43), cell shape (*p* = 0.18), or circumscription (*p* = 0.20). The only amelanotic tumor of our sample presented all the histological features of malignancy evaluated.

There was no association between histological classification and clinical features. All horses with solid coat colors carried melanomas and presented all the histological features of malignancy evaluated (vascular emboli, >10 mitosis per 10HPF, nuclear grade II to III). The majority of malignant tumors belonged to geriatric (n = 20 tumors) or adult horses that were 10 or more years old (n = 15 tumors).

### 3.3. Immunohistochemical Results

Regarding COX-2 final score, 33/77 (42.9%) tumors did not present any COX-2 expression, corresponding to 28/39 (71.8%) benign and 5/38 (13.2%) malignant tumors; 32/77 (41.6%) tumors presented low expression, corresponding to 8/39 (20.5%) benign and 24/38 (63.2%) malignant tumors; 12/77 (15.6%) presented high expression, corresponding to 3/39 (7.7%) benign and 9/38 (23.7%) malignant tumors. There was a statistically significant association between COX-2 score and histological classification (*p* = 0.001). According to Table 1, malignant tumors had considerably more positive expression, and benign tumors had more negative expression. It is also possible to observe that positive malignant tumors had mostly low expression (from the 33 positive malignant tumors, 24 (72.7%) had low expression, and 9 (27.3%) had high expression).

Regarding COX-2 classification (positive vs. negative), 44/77 (57.1%) tumors were positive, corresponding to 33/38 (86.8%) malignant and 11/39 (28.2%) benign tumors (Table 2). There was an association between COX-2 classification and histological classification (*p* < 0.001), with malignant tumors having a 16.8 times more likelihood of being positive for COX-2 expression. There was also an association between COX-2 classification and all the histological features of malignancy, namely mitotic count (*p* = 0.005) and intravascular emboli (*p* < 0.001), which means that COX-2 positive tumors had significantly more mitosis and more vascular emboli (Table 3 and Table 4 respectively). COX-2 and nuclear grade were also associated (*p* < 0.001). However, no association was seen with the other histological features evaluated, nor with any clinical feature.

**Figure 1:**
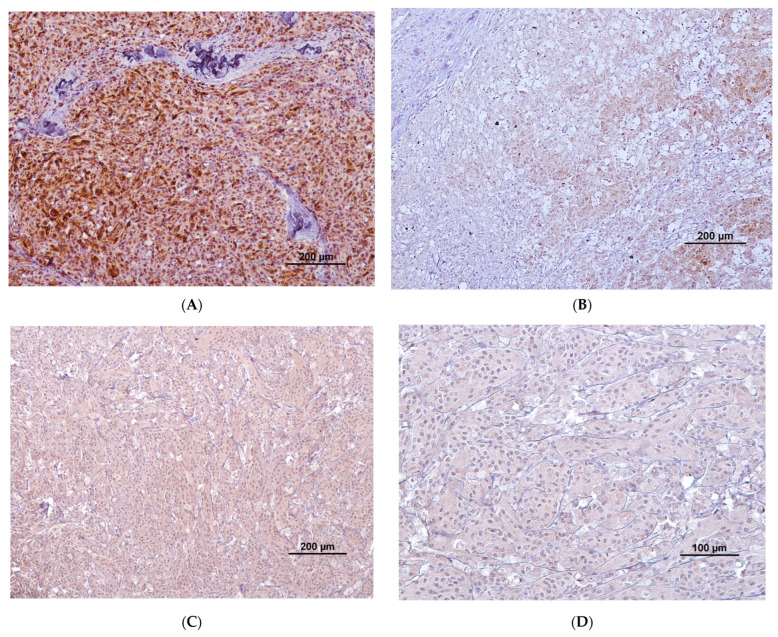
(**A**) COX-2 immunolabeling (brown staining) with high extension and strong intensity. Cytoplasmatic and perinuclear staining are present; (**B**) low extension of labeling with most tumoral cells being negative and some cells presenting weak-to-moderate staining in the cytoplasm and perinuclear region; (**C**) diffuse cytoplasmatic staining with moderate intensity; (**D**) cytoplasmatic staining with weak intensity.

Regarding COX-2 extent of labeling (Table 5), 6/77 (7.7%) tumors presented with more than 50% of labeled cells, corresponding to 6/38 (15.7%) malignant tumors; 26/77 (33.8%) tumors presented with between 21 and 50% of labeled cells, corresponding to 18/38 (47.4%) malignant tumors and 8/39 (20.5%) benign tumors; 11/77 (14.3%) tumors, presented with 6–20% of labeled cells, corresponding to 8/38 (21.1%) malignant tumors and 3/39 (7.7%) benign tumors; 1/77 tumors (1.3%) presented with 1–5% of labelled cells, corresponding to 1/38 (2.6%) malignant tumors. There was a statistically significant difference in the COX-2 extension medians of labelling between benign and malignant tumors (*p* < 0.001), with malignant ones presenting with a higher extension of labelling. The horse that had multiple melanomas had different extensions of labelling between each individual tumor. Regarding the horse with multiple melanocytomas, all the individual tumors were negative. Figure 1A,B represent different extensions and intensities of labeling in two different melanocytic tumors.

Regarding COX-2 intensity of labelling (Table 6), 11/77 (14.3%) tumors presented a strong intensity, corresponding to 8/38 (21%) malignant tumors and 3/39 (7.7%) benign tumors; 15/77 (19.5%) presented a moderate intensity, corresponding to 10/38 (26.3%) malignant tumors and 5/39 (12.8%) benign tumors; 18/77 (23.4%) presented a weak intensity, corresponding to 15/38 (39.5%) malignant tumors and 3/39 (7.7%) benign tumors. There was a statistically significant difference in COX-2 intensity medians of labelling between the benign and malignant melanocytic tumors (*p* < 0.001), with malignant tumors presenting a higher intensity of labelling. The horse that had multiple melanomas had different intensities of labelling between each individual tumor. Figure 1C,D present the moderate and weak COX-2 labeling intensities, respectively.

Regarding COX-2 localization, all tumor cells presented cytoplasmatic labelling, with a few also presenting perinuclear staining. COX-2 localization was not associated with any histological or clinical feature evaluated.

Results of COX-2 extent and intensity of labeling in cutaneous and mucocutaneous tumors are presented on Table 7 and Table 8, respectively. To summarize, 18/36 (50%) cutaneous and 8/30 (27%) mucocutaneous tumors were negative for COX-2, and 18/36 (50%) cutaneous and 22/38 (73%) mucocutaneous tumors were positive for COX-2. No association between COX-2 classification (positive/negative) and tumor location (cutaneous/mucocutaneous) was detected (*p* = 0.07). COX-2 extension and intensity medians of labelling did not differ between cutaneous and mucocutaneous location (*p* = 0.07 and *p* = 0.5, respectively).

## 4. Discussion

Even with the increasing prevalence of oncological diseases, the molecular features related to tumor initiation, maintenance, and progression are still poorly understood, especially regarding equine melanocytic tumors [9,47,48,49]. These tumors were long neglected because of their harmless behavior, justifying the scarce amount of research [15]. Thus, COX-2 expression in equine tumors is still poorly explored [22]. A comparison of immunohistochemical results between studies is sometimes difficult to perform due to differences in evaluation and classification methods [22], which does not allow for a full discussion of all articles regarding COX-2 in melanocytic tumors.

In 2008, Thamm et al. evaluated COX-2 expression in equine melanomas (n = 11), sarcoids (n = 14), and SCC (n = 37) [34]. Although no information was given regarding histological classification of these tumors, 64% were positive and 36% were negative for COX-2 expression. Compared with our entire sample, we found fewer positive tumors (57.1%). Although the authors of the above-mentioned study discuss the results of COX-2 in melanomas in a positive light, we believe that these results do not allow us to draw any different conclusions from those of our study. On the contrary, the results of the aforementioned study seem even less favorable. If we take a close look into the extension and intensities of labeling, these are lower than are those we found. They had more tumors presenting an extension of labelling between 1 and 20% labelled cells (45% vs. 15.6% in our sample), fewer tumors presenting 21–50% of labeled cells (18% vs. 33.8% in our sample), and no tumors presenting >50% of labeled cells (in contrast to 7.7% found in our sample). The same results occurred with intensity, where 54% of tumors of the above-mentioned study presented a weak-to-moderate intensity (we found it in 42.9% of tumors), and only 10% of tumors presented strong intensity, which is not considerably different from our 14.2%. Thus, in both studies, it seems that although equine melanomas tend to be positive for COX-2, their expression levels appear to be low. Regarding sarcoids, only 14% of tumors were positive. The results regarding COX-2 expression in SCC are not consistent. The aforementioned study found that 86% of SCCs were positive, and the extensions and intensities of labeling were higher than those in melanomas. Similar results of high COX-2 levels in equine SCC were found by Suárez-Bonnet et al. in 2018 [37]. However, other authors reported that most SCCs were negative for COX-2, underlining the importance of more studies in this field being conducted to corroborate or refute published results [35,36,39].

The role of COX-2 in canine melanocytic tumors and its association with other biomarkers has been better described [27,31,50,51]. In 2010, Pires et al. studied COX-2 expression in canine cutaneous, oral, and ocular melanocytic tumors [52]. The authors reported that 67.7% of malignant tumors were positive and that all benign tumors were negative. Although they indicated a lower proportion of positive melanomas than that in our study, they found more malignant tumors with >50% of labeled cells (25% vs. 15.7%). In 2004, Mohammed et al. analyzed 15 canine oral melanomas, of which 60% were positive [53]. None of the tumors presented weak intensity of staining, which is in contrast with our sample since 39.5% of malignant tumors evaluated had weak intensity. In 2020, Silveira et al. tested COX-2 expression in 85 canine malignant melanocytic tumors (29 oral and 56 cutaneous) [51] and found that 7% of oral and 12% of cutaneous tumors did not present COX-2 expression, with oral melanomas contrasting more with our findings (7% vs. 13.2%). We have already explored differences in COX-2 expression between melanomas of dogs and horses in a small sample of tumors, and we found statistically significant differences between them, with canine melanomas showed higher COX-2 levels [54].

In the present study, some benign tumors were positive for COX-2. This result contrasts with most studies in canine melanocytic tumors where all benign tumors were negative, although in 2016, Gregório et al. reported that some melanocytomas were weakly positive for COX-2 staining [27]. In the literature on humans, there are also reports of melanocytomas being positive for COX-2 as mentioned later in the Discussion. Some authors hold that the high cellular heterogenicity can explain how some tumor cells of melanocytomas present COX-2 expression [55]. Other authors have stated that both melanocytomas and melanomas can have COX-2 expression but at different levels, with expression increasing with disease progression and severity [56]. In the case of equine melanocytic tumors, there is a specific feature that can be related to COX-2 expression in benign tumors. In contrast to most canine melanomas that arise de novo, equine melanocytic tumors could have a time-dependent transformation of benign into malignant tumors [7]. The presence of COX-2 in benign tumors may promote this transition to malignancy. Although this malignant transformation is more common in horses, this hypothesis was also promoted by Nascimento et al. in 2016 [57], proposing that COX-2 levels found in melanocytomas of some studies [58] could be a sign of increased risk for malignancy. However, more evidence is needed to corroborate this hypothesis.

No association was found between COX-2 expression and tumor location in our sample. However, studies in dogs found significant differences between oral and cutaneous melanocytic tumors, with the former presenting higher levels of this biomarker [52,59]. There is a consensus that COX-2 is expressed differently between aggressive and nonaggressive tumors [31]. As such, it makes sense that canine oral melanocytic tumors present higher levels since these are considered much more aggressive than are the cutaneous forms. This supports the hypothesis that the low levels of COX-2 found on this work could be related to the less aggressive behavior of these tumors in horses.

Similarly to melanoma in dogs, human melanoma differs significantly from equine melanoma, being more aggressive and often being associated with a poor prognosis [60,61]. In 2016, Nascimento et al. found that all melanocytomas were negative and all melanomas were positive, with 92.3% presenting more than 50% of labeled cells, which is dramatically different from our results (15.7%) [57]. In 2017, Botti et al. reported that 93.3% of primary melanomas and 89% of metastatic melanomas presented COX-2 expression [25]. Again, the percentage of positive tumors is higher than that in our work, but the results underline the fact that some malignant tumors may not express COX-2. In 2019, Ghasemi et al. analyzed 45 melanocytic tumors (20 malignant and 25 benign) and found that 65% of malignant tumors presented strong intensity of COX-2 labeling and that 35% had moderate intensity of labeling, which contrasts with our work, where we found 21% with strong and 26.3% with moderate intensities of labelling [62]. Furthermore, Ghasemi et al. found 88% of melanocytomas to be positive for COX-2, which is far more than we reported. However, all benign tumors from the aforementioned article had weak labelling intensity, while 7.7% of our benign tumors had strong intensity.

One of our main findings is the statistically significant difference in COX-2 expression between benign and malignant melanocytic tumors. Melanomas were more often positive and presented higher extensions and intensities of labeling. Therefore, there was a significant association between COX-2 expression and the histological features of malignancy. These results are in close accordance with the literature regarding canine and human melanoma, with COX-2 also being associated with malignancy [27,62,63,64]. Even in the literature on humans, in which some studies reported that both melanocytomas and melanomas were positive, they found statistically significant differences in extension, intensity, and final scores [55,58,62]. Some of these studies suggest COX-2 to be a potential biomarker for the differentiation of benign and malignant tumors [55,58,62]. However, since in horses, some benign tumors can express this biomarker and some malignant tumors may not express it, we agree with the opinion of Kuźbicki et al. (2014) that although COX-2 may be used as an aid in the diagnosis, it may not be very reliable [55].

Regarding location of COX-2 labeling found in our work, it is consistent with the literature, in that cytoplasmatic labeling is the most common, with perinuclear and membranous staining also being reported [22,27,28,34,51,52,65,66].

The value of COX-2 as a biomarker to predict the tumor response against NSAIDs is still a point of debate since some of these drugs have antitumoral effects that are independent of COX-2. Nevertheless, a bulk of the literature show that NSAIDs or COX-2 selective inhibitors are more effective in tumors that express COX-2 [31,67]. Indeed, in the only case report where an NSAID (piroxicam) was used in a horse to treat SCC [40], there was complete remission with no relapse, and the immunohistochemical evaluation of the tumor revealed that 70% of tumor cells presented COX-2 with moderate-to-intense staining.

The absence of an association between COX-2 and all clinical and some histological features evaluated is in accordance with some studies [25,62,68] and in disagreement with others [27]. As previously stated, tumor location (oral vs. cutaneous) is a clinical feature that has some influence on COX-2 expression in canine melanocytic tumors. Although pigmentation is not fully considered a clinical/histological marker of aggressiveness, it has been reported in most species that amelanotic melanocytic tumors are often more aggressive. In 2020, Soares et al. also reported that amelanotic melanomas presented a significantly higher COX-2 intensity of labelling than did conventional melanomas [69]. Such a conclusion could not be drawn in our work due to the limited number of amelanotic melanomas and the absence of clinical history. However, the only amelanotic tumor in our sample presented all the histological features of malignancy and had high COX-2 expression, with more than 50% of labelled cells and strong staining intensity. The coat color was another feature that was not associated with malignancy. However, the literature reports that melanomas of nongray horses do not have the typical benign behavior described above, with the course of the disease being similar to that of dogs and humans [9,10]. Once again, the low numbers of nongray horses do not allow us to draw conclusions. Nevertheless, only one cremello horse presented high COX-2 expression, with more than 50% of labeled cells with strong intensity. The other cremello horse did not express COX-2, and both the brown and the buckskin horses presented low COX-2 levels. The only horse that had multiple malignant tumors presented different extensions and intensities of labelling between them, which points to the possibility that these tumors progress differently over time and may respond differently to NSAID therapy.

Changes in COX-2 expression have been associated with tumor progression, and part of the molecular reasons for this is the role of COX-2 in promoting epithelial-to-mesenchymal transition (EMT) [21,30,32]. Increasing levels of COX-2 can lead to a downregulation of E-cadherin, enabling tumor cell migration [31]. Atypically high levels of E-cadherin have already been found in equine malignant melanomas [45]. Taken together, these findings lead us to speculate that perhaps equine melanomas may maintain high levels of E-cadherin and low levels of COX-2 for a longer time, which may contribute to the maintaining of tumor mass stability and low invasiveness typical of these equine tumors. However, further studies are necessary to corroborate this speculation.

Immunotherapy is one of the major research areas in oncology [70,71]. PD-1/PD-L1 blockade is one of the most recently developed types of immunotherapy, and despite good results, this therapy is already facing some resistance [72,73,74]. Upregulation of COX-2 is one of the resistance mechanisms proposed [25,74]. The low levels of COX-2 found on equine melanocytic tumors may suggest that its role in the impairment of a future PD-1/PD-L1 therapy may not be as relevant as that in humans and dogs. This increases the interest in testing the effectiveness of this immunotherapy in equine melanocytic tumors, as PD-L1 expression has been reported for these tumors [75,76]. However, since a high percentage of the melanomas were found to be positive for COX-2, NSAIDs as an adjunctive treatment should be explored.

Regarding clinical information, most results are in accordance with the current literature. Most horses were gray, which can be attributed to the genetic trait related to the graying process also being the etiological factor for this disease in horses [13,16,49]. The majority of the horses were geriatric, underlining the higher prevalence of the disease in older horses, as stated by several authors [13,15]. Regarding breed, no conclusion could be made since most horses were purebred Lusitano. However, most authors stated that breed is not a risk factor, and the higher prevalence of melanocytic tumors reported in some breeds is related to high numbers of gray horses they have [12,15,60]. The literature does not consider ulceration to be a feature of malignancy in equine melanocytic tumors [13]. Our results also found no association between histological diagnosis and superficial ulceration. The few existing studies on equine melanocytic tumor histology reported the presence of multiple cellular shapes irrespective of histological diagnosis [3], which is in accordance with our findings.

In 2016, Nascimento et al. suggested that the role of COX-2 in tumor progression may vary according to its expression level (high or low) [57]. Given this, it would be interesting in further studies to evaluate how the different COX-2 levels in equine melanocytic tumors may contribute to different clinical courses of the disease.

The main limitations of the present work are the absence of more clinical information and follow-up, which would allow us to study associations between COX-2 levels and tumor progression and aggressiveness and thus evaluate the prognostic value of this biomarker. More in-depth studies are needed to determine the true biological value of this marker in equine melanoma. However, this work is a step forward in the understanding of the molecular differences that exist in equine melanomas and may contribute to explaining their intriguing clinical behavior compared with other species.

## 5. Conclusions

In this study, we found that most equine melanomas express COX-2, differing significantly from melanocytomas. However, the overall findings of the present work also allows us to conclude that most equine melanomas express low levels of COX-2, differing from most studies in humans and canine melanomas. This finding relates quite well with the typical benign behavior of these tumors in horses, characterized by expansive mass growth, low invasiveness, and rare metastasis. Since most tumors were positive for COX-2, the effectiveness of NSAIDs should be studied in further studies.

## Figures and Tables

**Table 1:** Distribution of COX-2 final score between benign and malignant tumors and in the total sample.

	Benign	Malignant	Total	*p*
**Negative**	28/39 (71.8%)	5/38 (13.2%)	33/77 (42.9%)	0.001
**Low**	8/39 (20.5%)	24/38 (63.2%)	32/77 (41.6%)	
**High**	3/39 (7.7%)	9/38 (23.6%)	12/77 (15.5%)	

**Table 2:** COX-2 classification between benign and malignant melanocytic tumors and in the total sample.

	Benign	Malignant	Total	*p*
**Negative**	28/39 (71.8%)	5/38 (13.2%)	33/77 (42.9%)	<0.001
**Positive**	11/39 (28.2%)	33/38 (86.8%)	44/77 (57.1%)	

**Table 3:** Distribution of mitotic count (0; <10 or >10 mitosis) and the presence/absence of mitosis between COX-2 negative and positive tumors.

	Mitotic Count						Mitosis Evaluation			
COX-2 Classification	0	<10	>10	Total	*p*	COX-2 Classification	Present	Absent	Total	*p*
Positive	32	3	9	44	<0.001	Positive	12	32	44	0.005
Negative	32	0	1	33		Negative	1	32	33	
Total	64	3	10	77		Total	13	64	77	

**Table 4:** Distribution of tumor vascular emboli (present/absent) between COX-2-positive and COX-2-negative tumors.

	Tumor Vascular Emboli			
COX-2 Classification	Present	Absent	Total	*p*
Positive	24	20	44	<0.001
Negative	3	30	33	
Total	27	50	77	

**Table 5:** Distribution of COX-2 extent of labelling between benign and malignant melanocytic tumors.

Immunohistochemistry		Benign (n = 39)	Malignant (n = 38)	Total (n = 77)	*p*
**Extension**	0 (negative cells)	28	5	33	<0.001
	1 (1–5% cells)	0	1	1	
	2 (6–20% cells)	3	8	11	
	3 (21–50% cells)	8	18	26	
	4 (>50% cells)	0	6	6	

**Table 6:** Distribution of COX-2 extent of labelling between benign and malignant tumors.

Immunohistochemistry		Benign (n = 39)	Malignant (n = 38)	Total (n = 77)	*p*
**Intensity**	0 (negative)	28	5	33	<0.001
	1 (weak)	3	15	18	
	2 (moderate)	5	10	15	
	3 (strong)	3	8	11	

**Table 7:** Distribution of COX-2 extent of labelling between cutaneous and mucocutaneous melanocytic tumors.

Immunohistochemistry		Cutaneous (n = 36)	Mucocutaneous (n = 30)	*p*
**Extension**	0 (negative cells)	18	8	0.07
	1 (1–5% cells)	0	1	
	2 (6–20% cells)	4	6	
	3 (21–50% cells)	13	10	
	4 (>50% cells)	1	5	

**Table 8:** Distribution of COX-2 intensity of labelling between cutaneous and mucocutaneous melanocytic tumors.

Immunohistochemistry		Cutaneous (n = 36)	Mucocutaneous (n = 30)	*p*
**Intensity**	0 (negative)	18	8	0.5
	1 (weak)	4	12	
	2 (moderate)	7	7	
	3 (strong)	7	3	

## Data Availability

No new data were created.

## Supplementary Materials

The following supporting information can be downloaded at https://www.mdpi.com/article/10.3390/vetsci11020077/s1: Figure S1: Positive control (equine kidney). Note COX-2 immunolabeling (brown staining) in the cells of *macula densa*.

## References

[1] Strashilov S., Yordanov A. (2021). Aetiology and Pathogenesis of Cutaneous Melanoma: Current Concepts and Advances. Int. J. Mol. Sci..

[2] Nishiya A.T., Massoco C.O., Felizzola C.R., Perlmann E., Batschinski K., Tedardi M.V., Garcia J.S., Mendonça P.P., Teixeira T.F., Dagli M.L.Z. (2016). Comparative Aspects of Canine Melanoma. Vet. Sci..

[3] Seltenhammer M.H., Heere-Ress E., Brandt S., Druml T., Jansen B., Pehamberger H., Niebauer G.W. (2004). Comparative Histopathology of Grey-Horse-Melanoma and Human Malignant Melanoma. Pigment Cell Res..

[4] Wong K., van der Weyden L., Schott C.R., Foote A., Constantino-Casas F., Smith S., Dobson J.M., Murchison E.P., Wu H., Yeh I. (2019). Cross-Species Genomic Landscape Comparison of Human Mucosal Melanoma with Canine Oral and Equine Melanoma. Nat. Commun..

[5] Vandyck H.H., Hillen L.M., Bosisio F.M., Van Den Oord J., Zur Hausen A., Winnepenninckx V. (2021). Rethinking the Biology of Metastatic Melanoma: A Holistic Approach. Cancer Metastasis Rev..

[6] Eddy K., Shah R., Chen S. (2021). Decoding Melanoma Development and Progression: Identification of Therapeutic Vulnerabilities. Front. Oncol..

[7] Resende L., Moreira J., Prada J., Queiroga F.L., Pires I. (2015). Current Insights into Canine Cutaneous Melanocytic Tumours Diagnosis. Melanoma—Current Clinical Management and Future Therapeutics.

[8] Schmid F., Brodesser D., Reifinger M., Forte S., Semp P., Eberspächer-Schweda M.C., Wolschek M., Brandt S., Kleiter M., Pratscher B. (2019). Canine Oral Primary Melanoma Cells Exhibit Shift to Mesenchymal Phenotype and Phagocytic Behaviour. Vet. Comp. Oncol..

[9] Phillips J.C., Lembcke L.M. (2013). Equine Melanocytic Tumors. Vet. Clin. North Am. Equine Pract..

[10] Cavalleri J.M.V., Mählmann K., Steinig P., Feige K. (2014). Aetiology, Clinical Presentation and Current Treatment Options of Equine Malignant Melanoma—A Review of the Literature. Pferdeheilkunde.

[11] Sullins K.E. (2020). Melanocytic Tumours in Horses. Equine Vet. Educ..

[12] Moore J.S., Shaw C., Shaw E., Buechner-Maxwell V., Scarratt W.K., Crisman M., Furr M., Robertson J. (2013). Melanoma in Horses: Current Perspectives. Equine Vet. Educ..

[13] Knottenbelt D.C., Patterson-Kane J.C., Snalune K.L. (2015). Melanocytic Neoplasms. Clinical Equine Oncology.

[14] Campagne C., Julé S., Bernex F., Estrada M., Aubin-Houzelstein G., Panthier J., Egidy G. (2012). RACK1, a Clue to the Diagnosis of Cutaneous Melanomas in Horses. BMC Vet. Res..

[15] Pimenta J., Prada J., Cotovio M. (2023). Equine Melanocytic Tumors: A Narrative Review. Animals.

[16] Pilsworth R.C., Knottenbelt D. (2006). Skin Diseases Refresher Melanoma. Equine Vet. Educ..

[17] Yi Z., Gao Y., Yu F., Zhu Y., Liu H., Li J., Murua Escobar H. (2023). Interventions for Treatment of Cutaneous Melanoma in Horses: A Structured Literature Review. Vet. Res. Commun..

[18] Poore L.A., Rest J.R., Knottenbelt D.C. (2013). The Clinical Presentation of a Mid-Tail Melanocytoma with Sudden Malignant Transformation in a Bay Irish Draught Gelding. Equine Vet. Educ..

[19] Knottenbelt D.C., Patterson-Kane J.C., Snalune K.L. (2015). Tumours of the Skin. Clinical Equine Oncology.

[20] Valentine B.A. (2003). The Spectrum of Equine Melanocytic Tumours. Equine Vet. Educ..

[21] Hashemi Goradel N., Najafi M., Salehi E., Farhood B., Mortezaee K. (2019). Cyclooxygenase-2 in Cancer: A Review. J. Cell Physiol..

[22] Doré M. (2011). Cyclooxygenase-2 Expression in Animal Cancers. Vet. Pathol..

[23] Mohsin N.U.A., Aslam S., Ahmad M., Irfan M., Al-Hussain S.A., Zaki M.E.A. (2022). Cyclooxygenase-2 (COX-2) as a Target of Anticancer Agents: A Review of Novel Synthesized Scaffolds Having Anticancer and COX-2 Inhibitory Potentialities. Pharmaceuticals.

[24] Pu D., Yin L., Huang L., Qin C., Zhou Y., Wu Q., Li Y., Zhou Q., Li L. (2021). Cyclooxygenase-2 Inhibitor: A Potential Combination Strategy with Immunotherapy in Cancer. Front. Oncol..

[25] Botti G., Fratangelo F., Cerrone M., Liguori G., Cantile M., Anniciello A.M., Scala S., D’Alterio C., Trimarco C., Ianaro A. (2017). COX-2 Expression Positively Correlates with PD-L1 Expression in Human Melanoma Cells. J. Transl. Med..

[26] Wang H., Tran T.T., Duong K.T., Nguyen T., Le U.M. (2022). Options of Therapeutics and Novel Delivery Systems of Drugs for the Treatment of Melanoma. Mol. Pharm..

[27] Gregório H., Raposo T.P., Queiroga F.L., Prada J., Pires I. (2016). Investigating Associations of Cyclooxygenase-2 Expression with Angiogenesis, Proliferation, Macrophage and T-Lymphocyte Infiltration in Canine Melanocytic Tumours. Melanoma Res..

[28] Carvalho M.I., Pires I., Prada J., Raposo T.P., Gregório H., Lobo L., Queiroga F.L. (2017). High COX-2 Expression Is Associated with Increased Angiogenesis, Proliferation and Tumoural Inflammatory Infiltrate in Canine Malignant Mammary Tumours: A Multivariate Survival Study. Vet. Comp. Oncol..

[29] Hodorogea A., Calinescu A., Antohe M., Balaban M., Nedelcu R.I., Turcu G., Ion D.A., Badarau I.A., Popescu C.M., Popescu R. (2019). Epithelial-Mesenchymal Transition in Skin Cancers: A Review. Anal. Cell. Pathol..

[30] Zhang X., Qu P., Zhao H., Zhao T., Cao N. (2019). COX-2 Promotes Epithelial-Mesenchymal Transition and Migration in Osteosarcoma MG-63 Cells via PI3K/AKT/NF-ΚB Signaling. Mol. Med. Rep..

[31] Silveira T.L., Pang L.Y., Di Domenico A., Veloso E.S., Silva I.L.D., Puerto H.L.D., Ferreria E., Argyle D.J. (2021). COX-2 Silencing in Canine Malignant Melanoma Inhibits Malignant Behaviour. Front. Vet. Sci..

[32] Pearlman R.L., Montes de Oca M.K., Pal H.C., Afaq F. (2017). Potential Therapeutic Targets of Epithelial–Mesenchymal Transition in Melanoma. Cancer Lett..

[33] Maekawa N., Konnai S., Asano Y., Sajiki Y., Deguchi T., Okagawa T., Watari K., Takeuchi H., Takagi S., Hosoya K. (2022). Exploration of Serum Biomarkers in Dogs with Malignant Melanoma Receiving Anti-PD-L1 Therapy and Potential of COX-2 Inhibition for Combination Therapy. Sci. Rep..

[34] Thamm D.H., Ehrhart E.J., Charles J.B., Elce Y.A. (2008). Cyclooxygenase-2 Expression in Equine Tumors. Vet. Pathol..

[35] Rassnick K., Njaa B. (2007). Cyclooxygenase-2 Immunoreactivity in Equine Ocular Squamous-Cell Carcinoma. J. Vet. Diagn. Investig..

[36] Smith K.M., Scase T.J., Miller J.L., Donaldson D., Sansom J. (2008). Expression of Cyclooxygenase-2 by Equine Ocular and Adnexal Squamous Cell Carcinomas. Vet. Ophthalmol..

[37] Suárez-Bonnet A., Willis C., Pittaway R., Smith K., Mair T., Priestnall S.L. (2018). Molecular Carcinogenesis in Equine Penile Cancer: A Potential Animal Model for Human Penile Cancer. Urol. Oncol. Semin. Orig. Investig..

[38] Jottini S., Cantoni A.M., Muzzoni E., Reppas G., Corradi A. (2009). Immunohistochemical Expression of Cyclooxygenase-2 (Cox-2) in Four Cases of Equine Mammary Gland Tumours. J. Comp. Pathol..

[39] van den Top J.G.B., Harkema L., Ensink J.M., Barneveld A., Martens A., van de Lest C.H.A., van Weeren P.R., Gröne A. (2014). Expression of Cyclo-Oxygenases-1 and -2, and Microsomal Prostaglandin E Synthase-1 in Penile and Preputial Papillomas and Squamous Cell Carcinomas in the Horse. Equine Vet. J..

[40] Moore A.S., Beam S.L., Rassnick K.M., Provost P. (2003). Long-Term Control of Mucocutaneous Squamous Cell Carcinoma and Metastases in a Horse Using Piroxicam. Equine Vet. J..

[41] Elce Y., Orsini J., Blikslager A. (2007). Expression of Cyclooxygenase-1 and -2 in Naturally Occurring Squamous Cell Carcinomas in Horses. Am. J. Vet. Res..

[42] McInnis C., Giuliano E., Johnson P., Turk J. (2007). Immunohistochemical evaluation of Cyclooxygenase Expression in Corneal Squamous Cell Carcinoma in Horses. Am. J. Vet. Res..

[43] Kumar D., Rahman H., Tyagi E., Liu T., Li C., Lu R., Lum D., Holmen S.L., Maschek J.A., Cox J.E. (2018). Aspirin Suppresses PGE2 and Activates AMP Kinase to Inhibit Melanoma Cell Motility, Pigmentation, and Selective Tumor Growth In Vivo. Cancer Prev. Res..

[44] Zhou P., Qin J., Li Y., Li G., Wang Y., Zhang N., Chen P., Li C. (2017). Combination Therapy of PKCζ and COX-2 Inhibitors Synergistically Suppress Melanoma Metastasis. J. Exp. Clin. Cancer Res..

[45] Pimenta J., Pires I., Prada J., Cotovio M. (2023). E-Cadherin Immunostaining in Equine Melanocytic Tumors. Animals.

[46] Queiroga F.L., Pires I., Lobo L., Lopes C.S. (2010). The Role of Cox-2 Expression in the Prognosis of Dogs with Malignant Mammary Tumours. Res. Vet. Sci..

[47] Knottenbelt D.C., Patterson-Kane J.C., Snalune K.L. (2015). The Challenges and Problems of Equine Oncological Practice. Clinical Equine Oncology.

[48] Mair T. (2014). Equine Veterinary Education Virtual Issue on Oncology. Equine Vet. Educ..

[49] Seltenhammer M.H., Simhofer H., Scherzer S., Zechner P., Curik I., Sölkner J., Brandt S.M., Jansen B., Pehamberger H., Eisenmenger E. (2003). Equine Melanoma in a Population of 296 Grey Lipizzaner Horses. Equine Vet. J..

[50] Pires I., Prada J., Coelho L., Garcia A., Queiroga F., Murph M. (2011). Tumor-Associated Macrophages (TAMs) and Cox-2 Expression in Canine Melanocytitc Lesions. Melanoma in the Clinic—Diagnosis, Management and Complications of Malignancy.

[51] Silveira T.L., Veloso E.S., Gonçalves I.N.N., Costa R.F., Rodrigues M.A., Cassali G.D., Del Puerto H.L., Pang L.Y., Argyle D.J., Ferreira E. (2020). Cyclooxygenase-2 Expression Is Associated with Infiltration of Inflammatory Cells in Oral and Skin Canine Melanomas. Vet. Comp. Oncol..

[52] Pires I., Garcia A., Prada J., Queiroga F.L. (2010). COX-1 and COX-2 Expression in Canine Cutaneous, Oral and Ocular Melanocytic Tumours. J. Comp. Pathol..

[53] Mohammed S.I., Khan K.N.M., Sellers R.S., Hayek M.G., DeNicola D.B., Wu L., Bonney P.L., Knapp D.W. (2004). Expression of Cyclooxygenase-1 and 2 in Naturally-Occurring Canine Cancer. Prostaglandins Leukot. Essent. Fat. Acids.

[54] Pimenta J., Prada J., Garcia A., Queiroga F., Pires I., Cotovio M. Comparison of Immunohistochemical Expression of Cyclooxygenase-2 (COX-2) between Canine and Equine Melanomas. Proceedings of the European Congress of Veterinary Pathology & Clinical Pathology.

[55] Kuźbicki L., Urban J., Chwirot B.W. (2014). Different Detectability of Cyclooxygenase-2 (COX-2) Protein in Standard Paraffin Sections and Tissue Microarrays of Human Melanomas and Naevi—Comparative Study. Pathol. Res. Pract..

[56] Kuzbicki Ł., Sarnecka A., Chwirot B. (2006). Expression of Cyclooxygenase-2 in Benign Naevi and during Human Cutaneous Melanoma Progression. Melanoma Res..

[57] Nascimento J., Carlos R., Delgado-Azañero W., Mosqueda Taylor A., de Almeida O.P., Romañach M.J.B.A.B. (2016). Immunohistochemical Expression of Cyclooxygenase-2 (COX-2) in Oral Nevi and Melanoma. J. Oral Pathol. Med..

[58] Kuźbicki Ł., Lange D., Straczyńska-Niemiec A., Chwirot B.W. (2012). The Value of Cyclooxygenase-2 Expression in Differentiating between Early Melanomas and Histopathologically Difficult Types of Benign Human Skin Lesions. Melanoma Res..

[59] Martínez C.M., Peñafiel-Verdú C., Vilafranca M., Ramírez G., Méndez-Gallego M., Buendía A.J., Sánchez J. (2011). Cyclooxygenase-2 Expression Is Related with Localization, Proliferation, and Overall Survival in Canine Melanocytic Neoplasms. Vet. Pathol..

[60] Deleon M. (2021). Cutaneous Melanoma: A Comparative Study Between Gray Horses, Canines, and Humans.

[61] Van Der Weyden L., Patton E.E., Wood G.A., Foote A.K., Brenn T., Arends M.J., Adams D.J. (2016). Cross-Species Models of Human Melanoma. J. Pathol..

[62] Ghasemi M., Afshar P., Sheidaei S., Moeini Y., Larijani L. (2019). The Role of Immunohistochemistry Expression of COX-2 in Differentiating Pigmented Benign and Malignant Skin Neoplasms. Med. J. Islam Repub. Iran.

[63] Goulet A.C., Einsphar J.G., Alberts D.S., Beas A., Burk C., Bhattacharyya A., Bangert J., Harmon J.M., Fujiwara H., Koki A. (2003). Analysis of Cyclooxygenase 2 (COX-2) Expression during Malignant Melanoma Progression. Cancer Biol. Ther..

[64] Jafarian A.H., Roshan N.M., Gharib M., Moshirahmadi V., Tasbandi A., Ayatollahi A.A., Ayatollahi H. (2019). Evaluation of Cyclooxygenase-2 Expression in Association with Clinical-Pathological Factors in Malignant Melanoma. Iran J. Pathol..

[65] Paglia D., Dubielzig R., Kado-Fong H., Maggs D. (2009). Expression of Cyclooxygenase-2 in Canine Uveal Neoplasms. Am. J. Vet. Res..

[66] Minami S., Lum C., Kitagawa K., Namiki T. (2011). Immunohistochemical Expression of Cyclooxygenage-2 in Melanocytic Skin Lesions. Int. J. Dermatol..

[67] Seo K.-w., Coh Y.-r., Rebhun R.B., Ahn J.-o., Han S.M., Lee H.-w., Youn H.-Y. (2014). Antitumor Effects of Celecoxib in COX-2 Expressing and Non-Expressing Canine Melanoma Cell Lines. Res. Vet. Sci..

[68] Iacono D., Cinausero M., Gerratana L., Angione V., Scott C.A., De Maglio G., Pizzolitto S., Di Loreto C., Puglisi F., Fasola G. (2018). Tumour-Infiltrating Lymphocytes, Programmed Death Ligand 1 and Cyclooxygenase-2 Expression in Skin Melanoma of Elderly Patients: Clinicopathological Correlations. Melanoma Res..

[69] Soares C.D., Hernandez-Guerrero J.C., de Andrade B.A.B., Romañach M.J., Mosqueda-Taylor A., Carlos R., Macedo M.R.S., de Almeida O.P., Jorge J. (2020). Comparative Expression of Cyclooxygenase 2 and Ki67 in Amelanotic and Conventional Oral Melanoma. Med. Oral Patol. Oral Cir. Bucal.

[70] Hanks B.A. (2022). The “Inside” Story on Tumor-Expressed PD-L1. Cancer Res..

[71] Tarone L., Giacobino D., Camerino M., Ferrone S., Buracco P., Cavallo F., Riccardo F. (2022). Canine Melanoma Immunology and Immunotherapy: Relevance of Translational Research. Front. Vet. Sci..

[72] Hossain S.M., Eccles M.R. (2023). Phenotype Switching and the Melanoma Microenvironment; Impact on Immunotherapy and Drug Resistance. Int. J. Mol. Sci..

[73] Shields B.D., Koss B., Taylor E.M., Storey A.J., West K.L., Byrum S.D., Mackintosh S.G., Edmondson R., Mahmoud F., Shalin S.C. (2019). Loss of E-Cadherin Inhibits CD103 Antitumor Activity and Reduces Checkpoint Blockade Responsiveness in Melanoma. Cancer Res..

[74] Chen S., McMiller T., Sankaran P., Kampta K., Topalian S. (2021). The COX-2 Pathway as a Mediator of Resistance to Anti-PD-1 Therapy. J. Immunother. Cancer.

[75] Pimenta J., Prada J., Pires I., Cotovio M. (2023). Programmed Cell Death-Ligand 1 (PD-L1) Immunohistochemical Expression in Equine Melanocytic Tumors. Animals.

[76] Ganbaatar O., Konnai S., Okagawa T., Nojima Y., Maekawa N., Minato E., Kobayashi A., Ando R., Sasaki N., Miyakoshi D. (2020). PD-L1 Expression in Equine Malignant Melanoma and Functional Effects of PD-L1 Blockade. PLoS ONE.

