# Supplementary material for: Cyclooxygenase-2 (COX-2) Expression in Equine Melanocytic Tumors
Source: Vet Sci. 2024 Feb 7;11(2):77. doi: 10.3390/vetsci11020077 (PMC10891553; doi:10.3390/vetsci11020077)
Supplement: Supplementary file 1 [file vetsci-11-00077-s001.zip › vetsci-2839785-supplementary.pdf]

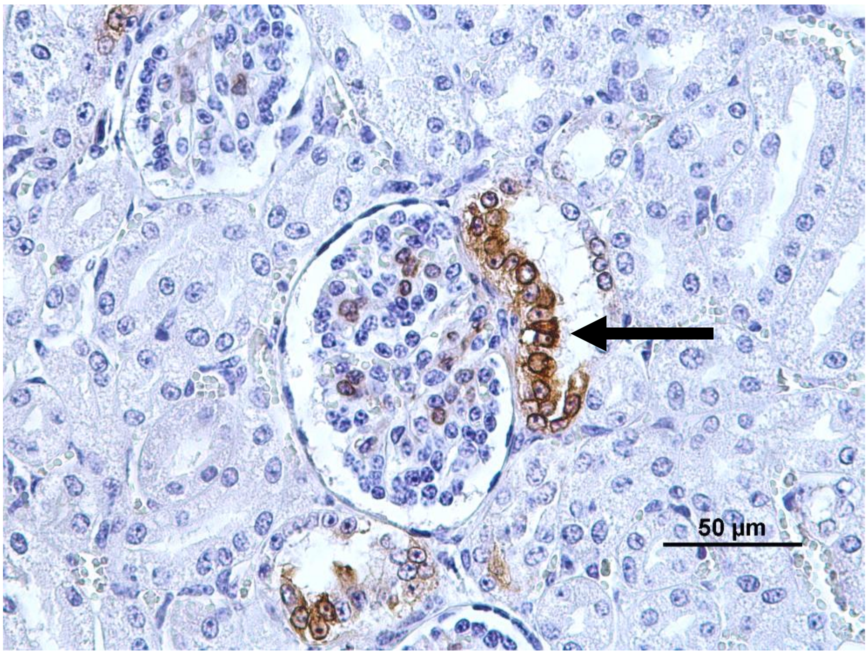


**Figure S1:** Positive control (equine kidney). Note COX-2 immunolabeling (brown staining) in the cells of *macula densa* (black arrow).
